# Reinforcing Collaboration and Harmonization to Unlock the Potentials of Advanced Therapy Medical Products: Future Efforts Are Awaited From Manufacturers and Decision-Makers

**DOI:** 10.3389/fpubh.2021.754482

**Published:** 2021-11-25

**Authors:** Tingting Qiu, Shuyao Liang, Yitong Wang, Claude Dussart, Borislav Borissov, Mondher Toumi

**Affiliations:** ^1^Département de Santé Publique, Aix-Marseille Université, Marseille, France; ^2^Faculté de Pharmacie, Université Claude Bernard Lyon 1, Lyon, France; ^3^Faculty of Public Health, Medical University, Sofia, Bulgaria

**Keywords:** public-private partnership, market authorization, health technology assessment, collaborations, advanced therapy medicinal products (ATMPs)

## Abstract

Some advanced therapy medicinal products (ATMPs) hold great promises for life-threatening diseases with high unmet needs. However, ATMPs are also associated with significant challenges in market access, which necessitates the joint efforts between all relevant stakeholders to navigate. In this review, we will elaborate on the importance of collaborations and harmonization across different stakeholders, to expedite the market access of promising ATMPs. Manufacturers of ATMPs should proactively establish collaborations with other stakeholders throughout the whole lifecycle of ATMPs, from early research to post-market activities. This covered engagements with (1) external developers (i.e., not-for-profit organizations and commercial players) to obtain complementary knowledge, technology, or infrastructures, (2) patient groups and healthcare providers to highlight their roles as active contributors, and (3) decision-makers, such as regulators, health technology assessment (HTA) agencies, and payers, to communicate the uncertainties in evidence package, where parallel consultation will be a powerful strategy. Harmonization between decision-makers is desired at (1) regulatory level, in terms of strengthening the international standardization of regulatory framework to minimize discrepancies in evidence requirements for market authorization, and (2) HTA level, in terms of enhancing alignments between regional and national HTA agencies to narrow inequity in patient access, and cross-border HTA cooperation to improve the quality and efficiency of HTA process. In conclusion, manufacturers and decision-makers shared the common goals to safeguard timely patient access to ATMPs. Collaboration and harmonization will be increasingly leveraged to enable the value delivery of ATMPs to all stakeholders.

## Introduction

The concept of advanced therapy medicinal products (ATMPs) was first introduced in the European Union (EU) Regulation EC No. 1394/2007 (1), which constituted a heterogeneous class of innovative medicinal products, such as gene therapy medicinal products, somatic cell therapy medicinal products, tissue-engineered products, and combined products (tissue or cell associated to a device) (2). The Committee for Advanced Therapies makes the scientific recommendations to European Medicines Agency (EMA) regarding whether or not the applied products satisfied the criteria for ATMPs and issues ATMP certification once the classification of the candidate was confirmed (3). Products that are manufactured using minimal manipulation processes (e.g., cell purification and washing), and are used for the homologous therapeutical purposes, are not regulated as ATMPs (3). ATMPs could be eligible for accelerated regulatory programs in the EU, such as orphan drug designations and Priority Medicine (PRIME) designation, if pre-defined criteria were satisfied. Notably, different terminologies were employed to define ATMPs in other countries (4). In the United States (US), according to the 21st Century Cures Act, some cell therapies, gene therapies, and tissue-engineering products could be qualified as regenerative medicine-advanced therapy (RMAT) designation if they are intended to treat, modify, reverse, or cure a serious or life-threatening disease or condition, and if preliminary clinical evidence indicates that the drug has the potential to address unmet medical needs for such disease or condition (5). The RMAT designation could benefit investigational drugs for more intensive interactions with Food and Drug Administration (FDA) and is eligible for accelerated programs, such as priority review and accelerated approval (5).

Advanced therapy medicinal products have revolutionized disease treatments and potentially brought new hopes for patients suffering from life-threatening diseases. However, compared to traditional medicines and biologicals, ATMPs faced significant difficulties in transiting scientific discovery to successful commercialization (6). To begin with, the manufacturing and quality control of ATMPs are complex processes, which must be carefully designed to guarantee quality, stability, consistency, and safety. For example, the isolation of viable cells from patients to obtain the cellular starting materials is time consuming, and the current process for vector production is poorly efficient (7). In the clinical development stage, reasonably sized, randomized, control trials (RCTs) seem infeasible for some ATMPs due to a small patient number, the absence of effective treatments to serve as active comparators, and the ethical controversy generated from assigning fragile patients to placebo when no effective treatment is available and ATMPs hold high promises (8).

In face of substantial uncertainties presented in the clinical evidence of ATMPs, decision-makers questioned extensively the durability of clinical benefits and the unforeseeable toxicities in the long run. In particular, health technology assessment (HTA) bodies criticized that limited evidence has deterred the robust assessments on the relative effectiveness and economic impacts of ATMPs (9). Regulators and HTA bodies therefore required that post-marketing studies must be conducted to bridge the evidence gaps in the initial submission. However, previous investigations indicated that the post-marketing scientific obligations were generally poorly fulfilled in terms of meeting completion deadlines and disappointing study quality (10). Moreover, despite no confirmatory evidence is available to demonstrate the “curative” benefits of ATMPs, manufacturers charged high prices with intentions to rapidly recoup the development investment (11). This made payers raise skepticism about the justification of the high prices of ATMPs in relation to the untransparent disclosure of manufacturing cost and often, paucity of clinical evidence (12).

Therefore, effective strategies to remove the aforementioned obstacles to enable facilitated patient access to promising ATMPs are urgently needed. Clearly, the successful market access of ATMPs could not be accomplished by one sole party, instead, it required intensive collaborations from all relevant stakeholders across different agencies and even across countries. In this review, we aimed to make strategical recommendations for manufacturers and decisions-markers by elaborating the importance of engaging all relevant stakeholders and enhancing harmonization to promote the timely market access of ATMPs.

This comprehensive review was conducted by searching PubMed database and Google scholar. We included English articles and official documents (e.g., guidelines, white papers, and reports) of regulators, HTA bodies, or other government organizations that were published from 2012 onward, because this was the year with the first ATMP, Glybera^®^, being approved in the EU (2). The combinations of following keywords were searched: (1) terms for products of interest: “ATMPs,” “cell therapy,” “gene therapy,” or “regenerative medicine,” (2) terms for activities of market access: “research and development,” “clinical trials,” “market authorization,” “regulation,” or “HTA,” and (3) terms for collaboration and harmonization: “partnership,” “involvement,” “engagement,” “cooperation,” “parallel consultation,” “joint activities,” or “cross borders.” The main focus of this review is EU-related regulations and activities, thus, the terminology of ATMPs was used in drafting this manuscript. Given that most ATMPs remain in the early stage of development, and only a limited numbers of ATMPs were currently approved, both manufacturers and decision-makers are in a learning process and are eager to explore the best approaches to enable faster patient access. This constitutes the larger challenge for ATMPs at this moment. We assumed that other stakeholders (e.g., patients) shared common desires to access ATMPs in a timely manner considering that several ATMPs are indicated for severe conditions without effective treatments. Therefore, we mainly target manufacturers and decision-makers in this review, while the roles of other stakeholders, such as patients and healthcare providers, were also described.

## Efforts From Manufacturers to Expand Collaborations of all Stakeholders

### Collaboration With Other Researchers and Developers

#### Public-Private Partnership

The previous study by Hanna et al. (2) suggested that the majority of trials investigating ATMPs were sponsored by non-commercial organizations (e.g., universities and hospitals), while it is notable that a large percentage of these trials were in the early stage of development (phase I or phase I/II). Complemented to these findings, Kassir et al. studied the gene therapy trials in the United States and observed that commercial organizations sponsored only 22% of phase I trials but over half of phase II trials and 100% of phase III trials (13). This implies the different roles that non-commercial and commercial organizations played in the ATMPs activities. Non-commercial organizations will lead the early research and development activities, while the pharma industry will resume the remaining responsibilities in the relatively late stage of clinical development and commercialization. Barriers for non-commercial parties to gain ownership of final inventions possibly included insufficient financial resources, potential liability issues, and lack of experience to navigate the complex regulatory and reimbursement process (14).

Cooperation between not-for-profit organizations and pharmaceutical companies or so-called “public-private partnership” is expected to complementarily harness the capabilities of each party, thus, providing great opportunities to ensure a faster and efficient “bench to bedside” transition (15). For example, the partnership between Novartis and the University of Pennsylvania in 2012 has contributed to the approval of the first chimeric antigen receptor (CAR)-T cell therapy, tisagenlecleucel (Kymriah^®^), in 2017. Public sectors are not restricted to academia, but also charity groups and government agencies. For example, Genethon, a French charity organization specialized in gene therapies for rare diseases, has established partnerships with several active Biotech companies and made contributions to the approval of onasemnogene abeparvovec (Zolgensma^®^) (16). NeuroNEXT initiative, funded by the US National Institutes of Health, has permitted industry access to their extensive resources related to rare neurological diseases (e.g., spinal muscular atrophy) through the Cooperative Research and Development Agreement mechanism (17). Furthermore, investigating the development histories of ATMPs approved in Europe, almost all of them were initially developed by non-commercial organizations, and license to pharmaceutical companies after the proof-of-concept was validated. The partnership agreements established and involvements of ‘Big pharma' that are relevant to approved ATMPs on the EU market are shown in Table 1.

**Table 1:** The collaborative mechanisms for ATMPs on the Europe market.

**Brand name**	**Developers**	**Involvements of non-profit organization**	**Major partnerships with other pharmaceutic companies**
Alofisel^®^	TiGenix (Belgium)	Spin-off from the Katholieke Universiteit Leuven and the Universiteit Gent.	- Alofisel was licensed to Takeda for the exclusive development and commercialization outside of the US (€15m) in 2016. - TiGenix was acquired by Takeda in 2018 for approximately €520m, representing €1.78 per share.
Strimvelis^®^	GSK (United Kingdom)	Originally developed in Milan by Ospedale San Raffaele (OSR) and Fondazione Telethon (Telethon). It was licensed to GSK through a strategic collaboration formed in 2010	- Transferred Strimvelis^®^ to Orchard Therapeutics in 2018. Under the agreement, GSK became an investor in Orchard Therapeutics, receiving a 19.9% equity stake along with a seat on the company's board. - MolMed signed an agreement with GSK for the manufacturing of virus vector of Strimvelis^®^ in 2011, 2013 and 2015.
Luxturna^®^	Spark Therapeutics (US)	Initially developed by University of Pennsylvania, in collaboration with Yale University, the University of Florida, and Cornell University. Spark entered into a licensing agreement with university of Pennsylvania in December 2014	- Spark Therapeutics entered into a licensing and supply agreement with Novartis in 2018, covering development, registration, and commercialization rights to Luxturna in markets outside the US. - Spark Therapeutics was acquired by Roche in 2019 for ~$4.3 billion, representing US$ 114.50 per share
Yescarta^®^	Kite Pharma (US)	Initially developed at the National Cancer Institute (NCI), Kite signed cooperative research and development agreement with NCI for the development and commercialization of KTE-C19 in 2013.	- Kite Pharma signed a strategic deal for development and commercialization of Yescarta^®^ in Japan with Daiichi Sankyo in 2017 - Kite Pharma was acquired by Gilead in 2017 for $11.9 billion, representing $180.00 per share - Kite Pharma entered a clinical trial collaboration with Pfizer to evaluate the combined therapy of KTE-C19 and utomilumab in 2018.
Kymriah^®^	Norvatis (Switzerland)	The treatment was originally developed by researchers at the University of Pennsylvania. Norvatis signed a global collaboration and licensing agreement in 2012 to further research, develop and commercialize of Kymriah^®^	- Oxford BioMedica signed agreements with Novartis to manufacture clinical grade material utilizing Oxford BioMedica's LentiVector^®^ gene delivery technology in 2013, 2017 and 2019. - Fraunhofer Institute for Cell Therapy and Immunology (IZI) signed agreements with Novartis to manufacture Kymriah^®^ in 2015 and 2018.
Zynteglo^®^	Bluebird bio (US)	The development of Betibeglogene autotemcel was initially led by the Marina Cavazzana (director of the biotherapy department at Necker-Enfants malades hospital) and Philippe Leboulch (head of the Institute of Emerging Diseases and Innovative Therapies of INSERM);	- Strategic manufacturing agreement with apceth Biopharma for the future European commercial production manufacturing of Zynteglo^®^ for transfusion-dependent β-thalassemia in 2016 - Lonza and bluebird bio entered into a strategic manufacturing agreement providing for the future commercial production of bluebird bio's Lenti-D™ and LentiGlobin™ drug products in 2016.
Zolgensma^®^	AveXis (US)	The intellectual property rights for Zolgensma were licensed from the University of Pennsylvania, Nationwide Children's Hospital (NCH), and Genethon in 2013	- AveXis acquired exclusive rights from REGENXBIO for NAV AAV9 vector for the development of treatments for SMA in 2014 - AveXis was acquired by Novatis for a total of $8.7b in cash, representing $218 per share in 2018.

*SMA, spinal muscular atrophy; US, United States; ATMPs, advanced therapy medicinal products*.

#### Partnership Agreements on Upstream and Downstream Activities

The personalized nature of ATMPs determined that massive challenges existed in both the upstream manufacturing activities (e.g., raw material sourcing, gene vectors supply, and sterility) and downstream administration activities (e.g., cross-border shipment, patient monitoring and tracking, and cryoconservation) (8). Unlike traditional medicines and biologicals, it seems impractical to expect that one company independently, either small-sized “Biotech” or “Big pharma,” will be sufficiently equipped to complete the whole “research-development-manufacturing-commercialization-tracking” process.

Instead, partnership agreements between multiple companies have emerged as important strategies to address the possible obstacles alongside each development step (18). Partnerships could be various in forms, including but not restricted to collaborative research and development agreements; joint venture; licensing agreements for a patent, advanced delivery vectors or cell culture; contract service providing manufacturing material, cold chain or tracking software; and commercialization contract concerning the commercialization and distribution of ATMPs in certain territories. Additionally, considering that ATMPs generally have complex structures (e.g., cell lines, virus vectors, and gene editing component), the developer of one ATMP may need to make several partnership agreements to acquire all the crucial technical skills required.

Looking deeper into the participating companies, innovation-driven “Biotech,” rather than risk-averse “Big pharma,” has emerged as major players in terms of advancing the research and development of ATMPs (19). The rationales behind the hesitations of “Big pharma” to involve when ATMPs were in the infancy stage seem obvious to understand. ATMPs development could be a venture business given the probably pessimistic market forecast of ATMPs for rare conditions, in contrast to the enormous resources required for research, manufacturing, testing, and monitoring of ATMPs. Fortunately, with the ATMPs field getting mature, the promises of ATMPs have attracted growing investments from “Big pharma” since 2010, either through forging partnership agreements or outright acquisition of leading “Biotech.”

#### Collaboration to Pool Resource and Knowledge

Improved knowledge on the genetic causes of rare diseases has boosted the development of orphan ATMPs to fulfill the high unmet medical needs of these life-threatening or debilitating diseases. However, the development of orphan drugs historically has been a frustrating journey, as reflected in more than 90% of rare diseases currently lacked effective treatments, despite fast scientific advancements, and a large number of clinical trials (17). One of the contributing factors is the small number of patients with a rare disease in a certain country, further compounded by that active researchers are geographically scattered. Another obstacle comes from that the large volume of pre-clinical and clinical trials data for rare diseases is not publicly accessible (20). Even worse, ATMPs will bring more complexities in addition to the challenges already faced by orphan drugs, which are largely contributed by their novel mechanisms of action, complicated manufacturing process, and insufficient knowledge on long-term efficacy and safety.

Therefore, if supported with appropriate protection for patient privacy and commercial confidentiality, clinical data sharing across multiple players hold great potentials to improve the quality of scientific research, inform better clinical designs, and facilitate the development of promising ATMPs targeting them (20). This could be achieved through establishing a global research network for rare disease to enhance the sharing of medical knowledge (e.g., natural history and genetic causes) and research infrastructures (21). Other advantages of international collaboration include the minimization of duplicative works and the mitigation of enrollment difficulties from small patient communities to benefit manufacturers (22).

For example, International Rare Diseases Research Consortium (IRDiRC) has established partnerships with other rare disease organizations, such as Orphanet in France and the Office of Rare Diseases Research in the United State. IRDiRC has contributed to broaden the accessibility of resources and investment and promote the harmonization of international standards for the classification/coding of rare diseases (23). Regarding ATMPs, the International Society for Cell and Gene Therapy, the International Society for Stem Cell Research, and Alliance for Regenerative Medicine could be perceived as pioneers to foster the collaborations among global communities that are dedicated to the ATMPs field. Clearly, manufacturers that are embracing collaborations and actively approaching such networks will be better positioned to keep close eyes on the adaptation of policies and remove the potential barriers. Moreover, building multi-national collaborations on the development of ATMPs for rare diseases would be of more significance in critical time, such as the current pandemic, when the research routine was severely disrupted, and patient recruitment (especially internationally) is more challenging (24).

Moreover, collaborations between multiple developers on clinical trials of ATMPs could be expected to increase in the future. Such collaboration could occur in two aspects: (1) developers are dedicated to investigating the combination of CAR-T cell therapies with checkpoint inhibitors or antibody therapies to enhance the effectiveness and durability of benefits in the treatment of hematological malignancy (Table 2) and solid tumors (25). Additionally, the use of immunosuppressive therapies (such as interleukin 6, IL-6, inhibitor and Tocilizumab) to decrease the incidence of CAR-T-related toxicity (e.g., cytokine release syndrome) is another focus of recent research (Table 2). (2) Increasing advancements in genomics and growing interests in precision medicine have promoted the movement toward the biomarker-guided clinical trials for oncology drugs, such as basket trials (one intervention to target multiple diseases), umbrella trials (one disease to be targeted by multiple inventions), and platform trials (multiple interventions against a common control group) under the master protocol framework. In general, such innovative study design is inherently more complex than conventional trials due to the inclusion of multiple patient cohorts or multiple interventions (26). While the adequate supports of a suitable screening platform for biomarkers (e.g., alterations in gene or molecule) will improve efficiency and expedite the clinical developments (27). Currently, the master protocol is still in infancy, there are many unanswered questions and challenges to address, so as to the feasibility of implementation in the ATMPs filed will be explored further (27). For example, as shown in the clinicaltrial.gov, no umbrella trials were identified for ATMPs, and only one basket trial (NCT02509507) for ATMPs, that investigates talimogene laherparepvec (Imlygic™) with a combination of pembrolizumab in several forms of hepatocellular carcinoma or non-hepatocellular carcinoma, is currently underway. Without doubts, the implementation of such innovative clinical trials strategy must be driven by the tight coordination between patient groups, pharmaceutical companies, and experts in genetics, translational science, and clinical research (28). Additionally, it is worth noticing that for companies adopting such study designs, they tend to include products only from their own portfolios. This highlighted that not-for-profit (e.g., academia and government) could play important roles in enhancing the participation across multiple companies and organizations, thus facilitating these innovative clinical trials and improve their quality (29).

**Table 2:** CAR-T cell therapies in combination with other products to improve efficacy or safety.

**CAR-T**	**Sponsors**	**Combination**	**MOA**	**Collaborators**	**Indication**	**Outcomes**	**Register No**.
Yescarta^®^	Kite, Gilead	Utomilumab	IgG2 mAb	Pfizer	Refractory large B-cell lymphoma	Efficacy; safety	NCT03704298
Yescarta^®^	Kite, Gilead	Lenzilumab	IgG1 k mAb	Humanigen, Inc.	R/R large B-cell lymphoma	Efficacy; safety	NCT04314843
Yescarta^®^	University of Washington	Acalabrutinib	BTK inhibitor	AstraZeneca; National Cancer Institute (NCI)	B-cell lymphoma	Efficacy; safety	NCT04257578
Yescarta^®^	Kite, Gilead	Rituximab	CD20 mAb	Several cancer centers and universities	Refractory large B-cell lymphoma	Efficacy; safety	NCT04002401
Yescarta^®^	Kite, Gilead	Atezolizumab	PD-L1 mAb	Genentech	Refractory DLBCL	Efficacy; safety	NCT02926833
Kymriah^®^	Novartis	Ibrutinib	BTK inhibitor	Peter MacCallum Cancer Centre	R/R mantle cell lymphoma	Efficacy; safety	NCT04234061
Kymriah^®^	Novartis	Ibrutinib	BTK inhibitor	Several cancer centers and universities	R/R DLBCL	Efficacy; safety	NCT03876028
Kymriah^®^	Novartis	Pembrolizumab	PD-L1 mAb	Several cancer centers and universities	R/R DLBCL	Efficacy; safety	NCT03630159
Yescarta^®^	Fred Hutchinson Cancer Research Center	Anakinra	IL-1 inhibitor	National Cancer Institute (NCI); Sobi	Prevention of cytokine release syndrome	Safety	NCT04359784
Yescarta^®^	Jazz Pharmaceuticals	Defibrotide	Single-stranded oligonucleotides	Several cancer centers and universities	Prevention of CAR-T associated neurotoxicity	Safety	NCT03954106
Yescarta^®^	Masonic Cancer Center	Dexamethasone /Simvastatin	Small molecule	University of Minnesota	Treatment of neurotoxicity	Safety	NCT04514029
Yescarta^®^	Massachusetts General Hospital	Anakinra	IL-1 inhibitor	Kite, A Gilead Company	CAR-T related neurotoxicity	Safety	NCT04150913
Yescarta^®^	M.D. Anderson Cancer Center	Anakinra	IL-1 inhibitor	National Cancer Institute (NCI)	Reduction of CAR-T toxicity in R/R large B-cell lymphoma	Safety	NCT04432506
Yescarta^®^ Kymriah^®^	Incyte Corporation	Itacitinib	JAK1 inhibitor	Several cancer centers and universities	Prevention of cytokine release syndrome	Safety	NCT04071366
Yescarta^®^ Kymriah^®^	Jonsson Comprehensive Cancer Center	Anakinra	IL-1 inhibitor	Not available	Prevention of neurotoxicity in R/R large B-cell lymphoma	Safety	NCT04205838
Kymriah^®^	University of Pennsylvania	Tocilizumab	IL-6 inhibitor	Children's Hospital of Philadelphia	Cytokine release syndrome	Safety	NCT02906371

*CAR, chimeric antigen receptor; DLBCL, diffuse large B-cell lymphoma; MoA, mode of action; R/R, relapsed or refractory*.

### Engagement With Patients and Healthcare Givers

#### Importance of Patient Involvement

Patient involvement should be leveraged throughout the development lifecycle of ATMPs. In the stage of clinical trials, early interactions with patient advocacy groups can provide benefits to deepen knowledge on the natural history of rare diseases, to reach the patient community quickly, in addition to optimize the design of clinical trials (30). As a result, patient engagement will generate financial values through facilitating enrollment and avoiding protocol deviations of clinical trials (31).

The engagement of patients in the regulatory process is not new and increasingly highlighted, for example, EMA has allowed patients to be members of scientific committees with equal voting rights, such as the Committee for Advanced Therapies (32). In the FDA, the Patient-Focused Drug Development program was launched in December 2020 to empower perspectives of patients to be captured and incorporated into the drug evaluation process (33).

In the HTA process, similar initiatives exist in some countries to enrich the context of HTA recommendations (34). For example, in National Institute for Health and Care Excellence (NICE) and US Institute for Clinical and Economic Review (ICER), patient involvement played advisory roles in every step of the evaluation process, from the selection of topics to be evaluated (priority setting), participation in the initial assessment, preparation of the draft recommendation (appraisal), and the dissemination of final decisions (35) (Table 3). As claimed by the Health Technology Assessment International, the involvement of patients in HTA will add advantages in terms of strengthening relevance, fairness, equity, legitimacy, and capacity building (36).

**Table 3:** Patient involvement in the HTA process.

**Country**	**HTA agency**	**Guideline or framework for patient involvement**	**Patient (group) role in HTA process**
pan-EU	EUNetHTA	Patient Input in Relative Effectiveness Assessments (May 2019)	• Collect patient inputs in the scoping phase to inform the development of population, intervention, comparators and outcomes (PICO)
UK-England	NICE	Patient and public involvement policy (November 2013)	• Patients can be involved directly in producing or promoting guidance, quality standards and other products as formal members of NICE committees and working groups. • Can also be involved in the NICE's work by commenting, through their organizations, on draft versions of guidance scopes and draft recommendations, and by submitting evidence.
UK-Scotland	SMC	Guide for Patient Group Partners (2017); Guide to the Ultra-Orphan Pathway (May 2019)	• Patient group to identify the priorities and preferences of patients and what the added value of a particular medicine maybe to them. • Patient group to identify important aspects of the medicine, that: may not be represented in the published literatures; quality of life or other outcome measures that may not be well captured in the clinical trials or other research studies; and may not be automatically understood by SMC. • One representative per submitting patient group is able to participate at the SMC committee meeting during discussions. Their role is to answer questions from committee members, relating to patient and career issues, and provide points of clarity relating to their submission, as required. • Patient group submissions and, when relevant, the output from a Patient and Clinician Engagement (PACE) meeting
US	ICER	Patient Participation Guide (October 2020)	• Scoping: Give early input on a new topic • Draft report: provide comments on the draft evidence report and draft voting questions • Attend a public meeting • Read final evidence report and meeting summary
Canada	CADTH	Guidance for providing patient input	• Patient group to provide feedback on the draft recommendation • Patient engagement fall under all three main categories of reviews, but is most useful for standard reviews (new drugs, drugs with new indications, and selected new combination products) and cell and gene therapy review
Australia	PBAC	Consumer Evidence and Engagement Unit (September 2019)	• Consumers can provide comments on the list of applications due for consideration at the Pharmaceutical Benefits Advisory Committee (PBAC) meeting • A formal targeted public consultation is conducted at the start of the Medical services advisory committee (MSAC) process for an application
Germany	G-BA	Patient Involvement Act	• Leading nationwide advocacy groups are entitled to take part in discussions and submit petitions, but not to vote. • The organizations currently entitled include that The German Council of People with Disabilities; The Federal Syndicate of Patient Interest Groups; The German Syndicate of Self-Help Groups; The Federation of German Consumer Organisations
France	HAS	Not available	• Public Involvement Council was established in 2019; No specific framework for patient involvement is available; Public involvement as a one of the priorities indicated in its Strategic Plan 2019–2024
Italy	AIFA	Not available	• Open AIFA program: Patient associations, representatives of civil society, the academic world, pharmaceutical companies and any other interested party can send a motivated request for participation in the meetings, which usually take place on a monthly basis, compatibly with the institutional commitments of the AIFA top management.
New Zealand	PHARMAC	Not available	• PHARMAC has updated their HTA framework in consideration of patient perspectives in July 2016, which gives a greater emphasis on quality of life of individual patients rather than cost-effectiveness.

*AIFA, Italian medicines agency; CADTH, Canadian agency for drugs and technologies in health; EU, European Union; EUNetHTA, European Network for Health Technology Assessment; G-BA, Federal Joint Committee; HAS, Haute Autorité de Santé; HTA, health technology assessment; ICER, Institute for Clinical and Economic Review; NICE, National Institute for Health and Care Excellence; PBAC, Pharmaceutical Benefits Advisory Committee; PHARMAC, pharmaceutical management agency; SMC, Scottish Medicines Consortium; UK, United Kingdom; US, United States*.

One important contribution from patient involvement in HTA is to provide patient-reported outcomes (PROs), which are increasingly emphasized by HTA agencies to understand the potential (direct and indirect) benefits being appreciated, and the potential harms being concerned by patients (32). Specifically, the relevance and quantification of additional values associated with ATMP remained controversial topics. Therefore, patient involvement can help to capture their opinions regarding whether they are willing to take risks for high uncertainties in exchange for a possibility of increased long-term survival (i.e., value of hope). Additionally, conflicting evidence is available in terms of the existence of patient preference toward potentially one-off ATMPs (e.g., gene replacement therapies) over regular treatments offering the same ‘total' health gains over many years (37). This is another field for further research that calls for the contributions of patients.

Whereas it should be recognized that patients expressed confusion and misconceptions about ATMPs, and they emphasized that more information related to the potential benefits, risks, and logistical requirements of clinical trials is paramount for them to make informed decisions (38). The Coronavirus Disease-2019 (COVID-19) crisis underscored the advantages of “decentralized clinical trials” that employ Telemedicine for patient monitoring and remote collection of follow-up data. More efforts must be in place to make sure that patients are familiar and comfortable with the remote approaches, thus guaranteeing the patient engagements and unbiased evidence collection are not compromised (39). Moreover, when utilizing the evidence directly collected from patients, how to improve its transparency, consistency, and credibility to properly incorporate them into HTA decision-making is worthy of further research (40).

#### Importance of Healthcare Providers Involvement

The management of ATMPs is a time-consuming process that required specialized personnel and tight coordination for patient monitoring before and after the injection (41). Moreover, insufficient experience in administrating ATMPs and the overestimation of the potential risks associated with ATMPs may adversely affect willingness of healthcare providers to adopt ATMPs (42). This necessities continuous training for physicians and nursing staff on the administration procedures and the management of severe adverse effects (e.g., cytokine release syndrome) (43).

It should be noticed that many inherited diseases targeted by ATMPs have disease onset in childhood, while pediatric patients may be incapable of correctly describing their symptoms and experiences. Under such circumstances, clinician-reported outcomes (CliniRO) could serve as a reliable surrogate endpoint to PRO in the clinical trials (44). Additionally, clinical experts can also contribute to optimizing the economic models, which is reflected in providing insights on appropriate input parameters where published data are lacking, and in securing the representativeness of key model assumptions to clinical practice (45). Involvement of physicians in the HTA process of ATMPs is also emphasized, for example, in the newly released Canadian Agency for Drugs and Technologies in Health (CADTH) review process for cell and gene therapies in Canada (46). This is due to the complexity associated with the diseases targeted by ATMPs and the unique challenges in accommodating ATMPs into the current health system.

In the post-launch phase, manufacturers should work closely with healthcare providers to ensure that appropriate and adequate infrastructures for long-term evidence collection are established. This is one critical factor impacting claimed clinical benefits of ATMPs could be observed in the real-world setting (47). Likewise, the implementation of outcome-based payment will also be dependent on the active engagements of physicians, as illustrated in Netherland's experience that the compliance of low physicians to the protocol comprised one of the reasons for the discontinuation of alternative payments models (48). Therefore, the implication is that training healthcare providers on the standard process of post-launch collection relevant for HTA re-assessment are essential.

### Interactions With Regulators, HTA Bodies, and Payers

#### Communications With Regulators

As shown in the survey by Ten Ham et al. manufacturers considered regulatory hurdles as one of the biggest challenges (34% of response) hampering successful clinical translation and commercialization of ATMPs. The regulatory challenges that are most mentioned included requirements for submission pathways, pre-submission interactions, and product logistics issues (19). This suggests the importance of increasing regulators-manufacturers collaborations to provide more guidance and clarifications on the regulatory requirements specific to ATMPs issues.

In addition, regulators tended to show some degrees of flexibility in the evidence assessment of ATMPs (4). Manufacturers are encouraged to approach regulators at the earliest possible to discuss the potential difficulties in the clinical trials and consult their acceptability of alternative study design (e.g., historical comparison). Moreover, a variety of accelerated approval programs (exclusively or non-exclusively to ATMPs) (Table 4) are being implemented by regulators to streamline the market authorization of innovative products. Early interactions with regulators to discuss the eligibility to accelerated programs will be advantageous for manufacturers: once accepted, more frequent dialogues to gain more immediate expert advice from regulators will be allowed. This will enable manufacturers to be better prepared to build development strategies and potentially lower the possibility of future regulatory objections (49).

**Table 4:** Accelerated approval programs allow enhanced interactions between manufacturers and regulators.

**Regions**	**Programs**	**Eligible criteria**	**Consultation or interactions with regulators**	**ATMPs approved(Approval date)**
European Union	Orphan drug designation	• Intent for diseases that are life-threatening or chronically debilitating • Prevalence of condition is not more than 5 in 10,000 in the EU • No satisfactory treatments are available; or medicine of significant benefits to patients	• Scientific advice specifically for orphan medicines called protocol assistance. • Administrative and procedural assistances for micro, small and medium-sized enterprises	Holoclar^®^ (17/02/2015)Strimvelis^®^ (26/05/2016)Alofisel^®^ (23/03/2018)Kymriah^®^ (22/08/2018)Yesccarta^®^ (23/08/2018)Zyteglo^®^ (29/05/2019)Zolgensma^®^ (18/05/2020)Tercartus^®^ (14/12/2020)Libmeldy^®^ (17/12/2020)
	Priority medicine (PRIME) designation	• Offer a major therapeutic advantage over existing treatments • Show its potential to benefit patients with unmet medical needs based on early clinical data	• Appoint a rapporteur from CHMP or CAT • Intensive guidance on the overall development plan and regulatory strategies • Scientific advice at key development milestones, involving additional stakeholders, such as HTA body	Kymriah^®^ (22/08/2018)Yesccarta^®^ (23/08/2018)Zyteglo^®^ (29/05/2019)Zolgensma^®^ (18/05/2020)Tercartus^®^ (14/12/2020)
United State	Fast track	• Indicated for serious conditions • Fill an unmet medical need defined as providing a therapy where none exists or providing additional benefits than available therapy (such as superior effectiveness or avoiding serious side effects)	• More frequent meetings with FDA to discuss the drug's development and data collection plan • More frequent written communications with FDA to discuss the clinical trials design and use of biomarkers	Provenge^®^ (29/04/2010)Hemacord^®^ (10/11/2011)Imlygic^®^ (27/10/2015)Zolgensma^®^ (24/05/2019)Ryplazim^®^ (04/06/2021)
	Breakthrough designation	• Intend for serious condition • Preliminary clinical evidence indicates that the drug may demonstrate substantial improvement over available therapy on a clinically significant endpoint	• All Fast Track designation features • Intensive guidance on an efficient drug development program, beginning as early as Phase I • Organizational commitment involving senior managers	Kymriah^®^ (30/08/2017)Luxturna^®^ (18/12/2017)Yescarta^®^ (18/10/2017)Zolgensma^®^ (24/05/2019)Breyanzi^®^ (05/02/2021)Tecartus^®^ (24/07/2020)Abecma^®^ (26/03/2021)
	Regenerative Medicine Advance therapy (RMAT) designation	• Regenerative medicines that are intended for a serious or life-threatening disease or condition • Preliminary clinical evidence indicates that the drug has the potential to address unmet medical needs for such disease or condition	• All Breakthrough designation features • Early interaction to discuss potential surrogate or intermediate endpoints	Breyanzi^®^ (05/02/2021)Stratagraft^®^ (15/06/2021)
	Rare pediatric Disease Designation	• Rare disease and one where the disease is serious or life-threatening with the serious or life-threatening manifestations primarily affecting individuals from age zero to 18	• Protocol assistance: different types of meetings that the Agency encourages, and these include pre-IND meetings, end-of-Phase I meetings, end-of-Phase II meetings, pre-NDA meetings, and then there is also a number of meetings that are conducted during the course of the review of the marketing application.	Kymriah^®^ (30/08/2017)Luxturna^®^ (18/12/2017)Zolgensma^®^ (24/05/2019)Ryplazim^®^ (04/06/2021)
Japan	SAKIGAKE designation	• Product innovativeness • Intent for a serious or life-threatening condition • Significantly improvement in effectiveness or safety compared to existing treatments • Develop the product rapidly and • file an application for approval in • Japan, ahead of other countries	• Consistent prioritized consultation • Pre-application consultation • Assigning a PDMA manager as a concierge.	STR01 (28/12/2018)Stemirac^®^ (12/11/2018)Zolgensma^®^ (26/02/2020)
	Orphan regenerative medical product	• Prevalence of the disease is <50,000 patients in Japan • Indicated for serious disease with high unmet needs.	• PMDA provide advices and consultations concerning the interpretation of designation criteria and other regulatory matters.	JACE (29/09/2016)Temcell^®^ (18/09/2015)Zolgensma^®^ (26/02/2020)

*CAT, Committee for Advanced Therapy; EU, European Union; FDA, Food and Drug Administration; HTA, health technology assessment; IND, investigation new drug; NDA, new drug application*.

#### Communications With HTA Bodies and Payers

Prior to embarking on pivotal trials, manufacturers are advised to engage with HTA bodies to ensure that evidence requirements for reimbursements have been sufficiently factored into the development plan (50). EU experiences provide some implications why this is imperative: HTA and reimbursement decisions are made at the discretions of an individual country, despite that centralized market authorization permitted licensing approval across all Member States simultaneously.

For example, NICE utilized an explicitly defined incremental cost-effectiveness ratio threshold to evaluate if the investigated products are acceptable for reimbursement, while Haute Autorité de Santé in France and Gemeinsame Bundesausschuss in Germany stressed the added clinical benefits against alternatives for positive recommendations. Such divergence could be more paramount for ATMPs due to substantial complexity arising from their novelty, clinical promises, and uncertainty in clinical evidence and affordability challenges. Therefore, conversations with HTA bodies in the individual country to enable a tailored “reimbursable evidence dossier” in place could be meaningful to secure patients access, and commercialization of ATMPs will not experience a significant delay in a given country (50). This is also applicable to fragmental markets where exist multiple payers (e.g., US), or where the HTA decisions were made at the regional level (e.g., Sweden), suggesting that appropriate dossiers target the specific evidence requirements of each payer segment should be prepared (51).

Additionally, payers have increasingly adopted alternative payment strategies, such as performance-based payment or installment payment, to strike the balances between the clinical benefits shown in real-world setting and sustainability of affordability (52). A partnership between manufacturers and payers is necessary to reach the agreements regarding outcomes to be collected for the evaluation of long-term effectiveness and comparative advantages (53). Collaborations between them are also needed in terms of clarifying the definition of clinical milestones, the payment amount per installment, the timeframe for payment execution, and the methods to adjust the payments based on the achieved clinical outcomes (54). In general, manufacturers and payers shared common interests to promote the timely market access of ATMPs, while collaboration to reach agreements mutually beneficial to both parties will be the key to achieve it.

#### Parallel Consultation With Regulators and HTA Bodies

In addition, it is important to bear in mind the possible discrepancies in the evidence requirements imposed by regulators and HTA bodies. Regulators focus on efficacy and safety evidence to assess the benefit-risk of applicants (internal validity), while payers focus on the relative effectiveness, safety, and (possible) cost-effectiveness to assess the additional benefits over alternatives (external validity) (9). Accordingly, regulators showed more favorable attitudes toward the market approval of ATMPs despite immature evidence, in contrast to HTA bodies hesitated to endorse ATMPs due to substantial uncertainties. Moreover, the implementation of expedited programs (e.g., accelerate approval and conditional market authorization) could further widen the divergence between regulators and HTA bodies, given that less comprehensive evidence will be acceptable by regulators if benefits overweighted the potential risks (55). Such discrepancies could partly contribute to the commercial failure of early-approved ATMPs in the EU, such as Glybera^®^ and Provenge^®^, both were withdrawal from the market because “not-reimbursed” status significantly restricted patient affordability and clinical adoption.

Therefore, there is an urgent call for enhancing conversations between regulators and HTAs, to ensure that (1) evidence uncertainties (e.g., surrogate outcomes and indirect comparison) associated with ATMPs have been sufficiently communicated (9), (2) agreements are reached regarding the qualifications of drugs for expedited approval programs based on the “unmet clinical needs” criterion, and (3) the requirements for post-launched evidence collection will meet the expectations of both parties (56).

For example, in the EU, the adaptive pathways approach, which was a pilot project from 2014 to 2016, provided a framework for parallel scientific advice with both HTA bodies and regulators. Two ATMPs, bluebird bio's LentiGlobin BB305 (approved as Zynteglo^®^) and Pluristem Therapeutics' PLX-PAD, were enrolled in the adaptive pathways program (3). Subsequently, parallel consultations with EMA and the European Network for Health Technology Assessment (EUnetHTA) were initiated in July 2017, which allowed developers to obtain simultaneous feedbacks on the evidence requirements for MA and reimbursement (57). This will empower developers to be better informed before embarking on the clinical trials, thus improving the chance of acceptance from both parties and speeding up the ultimate market access (58). Although the number of products selected for parallel consultations will be limited due to resource constraints, we consider ATMPs will still likely be qualified for this process because (1) ATMPs will meet the eligibility criteria for parallel consultation in terms of bringing added benefits for patients by a new mode of action, targeting life-threatening or chronically debilitating diseases, or responding to unmet needs; (2) EUnetHTA claims that selected products should represent a wide array of topics, such as orphan drugs and ATMPs; and (3) previous studies showed that biologicals accounted for a large percentage of all products that have undergone parallel consultation (59).

## Efforts From Decision-Makers to Enhance International Harmonization

### Harmonization at Regulatory Level

#### Hospital Exemption Rules in European Countries

At the EU level, with the objective to facilitate patient access to ATMPs for diseases with urgent medical needs, so-called “hospital exemptions (HE)” empowered each Member State to provide unauthorized ATMPs in their jurisdiction under exceptional circumstances. To be accepted for HE, ATMPs must be customized-made, prepared to comply with specific quality standards, used in the hospital settings on a non-routine basis, and administrated under the exclusive responsibility of a medical practitioner for the individual patient (60). However, interpreting the definition (e.g., non-routine use) and the qualification criteria for HE rules varied significantly across different Member States. Some required that clinical data must be submitted prior to a HE license, while some others may grant it in the absence of any preclinical or clinical data (61). Such inconsistencies in the regulations of HE were scrutinized for jeopardizing the stringency of centralized market authorization and creating unfair competition for manufacturers of ATMPs, who made substantial investments in conducting clinical trials and addressing regulatory challenges (3). Therefore, better harmonization of the HE regulation across the EU is urgently needed. This could be achieved by issuing EU-wide regulatory guidelines to outline the minimal quality, preclinical, and clinical data that are required for the grant of HE license (61).

#### Environmental Risk Assessment

The requirements of conducting environmental risk assessment (ERA) are mandatory for medicines containing genetically modified organisms (GMOs), aiming to examine their potentially harmful effects on the ecosystem and human health (62). Gene therapy vectors, genome editing therapy, and somatic cell therapies whose genetic material has been manipulated will normally be classified as GMOs, while the judgments are made on a case-by-case basis and could vary depending on the interpretations around the environmental and biosafety aspects of GMO-based medicines in different countries (62, 63). At the EU level, although the ERA submission for market authorization application is centralized and reviewed by EMA, the REA submission for clinical trials application is processed by national health authorities in each Member State. However, Iglesias-Lopez et al. observed that there was wide misalignment on the methods for risk assessment, documents to be sent, the timeline for submission, and the procedures to be followed (64). Divergent requirements will demand different measurements for the protection of patients and the environment, which will lead to variations in study protocols and add more complexity to conducting multinational clinical trials (63). Harmonization at the EU level is needed to uniform the terminology, classifications and requirements of ERA to improve efficiency, in which a single EU document and coordination of a parallel review between the different Member States (coordinated by sponsors) seems to offer advantages to address the divergencies (64).

#### Post-launch Patient Registries

Considering that ATMPs were generally approved based on less comprehensive evidence derived from single-arm trials with a small patient number and surrogate endpoints, post-launch evidence collection via patient registries could be powerful to bridge the evidence gap at the initial submission. However, for the patient register to succeed, it must be implemented in a rigorous and coordinated manner (65). Despite potential benefits, patient registries also faced several challenges, such as the poor data quality, non-inclusion of clinically relevant endpoints (e.g., patient satisfaction and quality of life), and the nature of non-comparative trials preventing the sound conclusion on relative effectiveness (66). Moreover, the misalignment in the requirements for post-launch evidence across European countries may create inconsistencies in the source of information used and the outcomes collected, thus undermining the efficiency of post-launch evidence collection. Therefore, coordinating data collection by establishing interoperable patient registries across multiple countries could be a useful strategy to reinforce harmonization, and possibly allow subsequent attempts for evidence aggregation and meta-analysis (37, 54).

The patient registries for ATMPs should ideally be international or based upon internationally agreed standards on data elements to be collected and terminology to be used. While, on the other side, it needs to be designed in an adaptive model that allows the collection of customized data in accordance with the local evidence requirements in different countries (67). For example, EMA has launched the initiative for patient registries since September 2015, which seeks to create an EU-wide framework to facilitate collaboration and harmonization within European countries. This contributed to positive opinions on two registries as a suitable platform for post-launch evidence collection: European Cystic Fibrosis Society Patient Registry, and European Society for Blood and Marrow Transplantation for CAR-T cell therapies (68).

As claimed by McGrath et al. (67), global registries for CAR-T cell therapies would provide benefits in the following aspects: (1) avoid siloed data collected in the private register by isolated market authorization holders and disease-focus group; (2) enable the comparison of safety, effectiveness, and cost-effectiveness between different CAR-T cell therapies; and (3) enable the comparisons of CAR-T against alternative interventions, such as bispecific molecules and stem cell transplantation. One of the fields that attracted substantial investments was gene therapies for hemophilia. In collaboration with multiple hemophile foundations across countries, a World Federation of Hemophilia Gene Therapy Registry is under construction, with the ambition to provide a large size, standardized, robust, and validated data collection platform available for all healthcare providers and patients (69). Global registries for ATMPs targeting other therapeutical areas should be explored, at least for those ATMPs with multiple players actively engaged.

#### International Regulation for ATMPs

Despite significant advancements in the development of ATMPs, there still lacks international standards clarifying the definition or classification of ATMPs. For example, when comparing the regulatory terminology and classifications of ATMPs, differences were indicated between FDA and EMA: cord blood is regulated as cellular therapy in FDA but not in EMA (4); human tissue products that contain living cells (e.g., skin replacement) could be classified as medical devices in FDA, but are classified as drugs in EMA (70). Discordances are also shown in the requirements for manufacturing and clinical trials of ATMPs (5). For example, Banda et al. suggested that EMA may employ more stringent requirements on good manufacturing practice earlier on compared to FDA (71). Additionally, the requirements for ERA assessment varied in terms that the applications for clinical trials and market authorizations for certain gene therapies could be excluded or exempted from ERA in the United States, while a full ERA will be required in the EU (63, 64). Consequently, inconsistent regulation of ATMPs makes the multicenter clinical trials across countries difficult to perform, which increases the administrative burdens of manufacturers to navigate the regulatory activities in different countries (72). It is unknown how such difference will translate into disparate approval decisions, while with a growing number of ATMPs entering the regulatory review process, it is questionable if the approval gap between different countries will be evidently widened.

Another challenge arising from the absence of international standards for ATMPs is that non-authorized ATMPs with uncertain efficacy and safety could be used in private clinics (73) in countries where stringent oversight on ATMPs is lacking. This will possibly trigger the “treatment tourism” of patients to foreign countries for receiving ATMPs that are not authorized in their own countries (38). However, the long-distancing travel will exhaust already vulnerable patients, in addition to the potential safety risks and high expenses of unauthorized ATMPs treatments.

Therefore, it could be meaningful to establish international coordination on ATMPs regulation to uniform the terminology, to standardize the criteria for ATMPs that are exempted from market authorization, and to clarify the minimal requirements of the dataset needed for the initiation of clinical trials and market authorization (74). Although one-size-fits-all regulation is unrealistic, it could at least help minimize the ambiguity and avoid conflicts in the regulatory requirements. Additionally, it might largely prevent “medical tourism” when the administration of unauthorized ATMPs will be supervised under the universal regulatory framework (4).

Regulators have already committed to enhance the communications between each other, such as the ATMPs cluster between FDA, EMA, and Health Canada, which is a forum for regularly discussing regulatory approaches, document exchanges, and guideline sharing related to ATMPs (74). International Coalition of Medicines Regulatory Authorities, as a voluntary network for global regulators, has promptly acted to improve the policy alignments during the COVID-19 pandemic, with more than 30 meetings being held during 1 year of outbreak. In the future, if such broad collaboration between regulators could expand into ATMPs filed, greater transparency, and clarification on ATMPs regulation could be expected (75).

### Harmonization at HTA and Reimbursement Level

#### Collaboration Between National and Regional HTA Organizations

In the United States and some European countries, HTA and reimbursement decisions are made at the regional level rather than at the national level. This means that one product that gets reimbursed in one region could fail to be reimbursed in another region. The disparity across different regions might be more evident in the case of ATMPs because their high price will raise financial challenges for less-resourced regions.

To mitigate unequal access to ATMPs caused by de-centralization in certain European countries, the European Organization for Rare Diseases (EURORDIS) recommended to centralize the HTA process of ATMPs in Sweden, Italy, and Spain to improve consistency at the regional level. Country-specific strategies to reducing the budget burdens at the regional level were proposed, such as (76) to establish state funding for ATMPs in Sweden, to involve regional payers in designing national outcome-based deals to align negotiation requirements in Italy, and to expand the use of national strategies, Valtermed (a risk-sharing agreement that included 3 ATMPs-Alofisel^®^, Yescarta^®^ and Kymriah^®^ as polit products), to other ATMPs in Spain. To make centralized HTA and reimbursement decision-making happen, collaborations between national and regional agencies must be highlighted to adjust current legislations for implementing them.

#### Joint HTA Activities Across European Countries

In the past decade, growth in the multicounty collaborations in aspects of HTA, price negotiation and drug procurement were observed in European countries. Such cohesive efforts were largely driven by the fact that the ever-increasing price of innovative drugs has burdened the healthcare system, especially for less developed countries or countries with small population. Instead of working in insolation, joint work across borders could potentially improve the efficiency of HTA, increase the price transparency, and enable greater affordability of expensive drugs through greater bargaining power.

At the pan-EU level, a legislative proposal of EU cooperation on HTA was issued in January 2018, in which joint clinical assessments, joint scientific consultations, the identification of emerging health technologies, and voluntary cooperation were identified as four pillars (77). In response to the call, EUnetHTA, now consisting of 81 organizations from 29 countries, is committed to improve the quality and efficiency of joint HTA work across European countries. Up to now, seven ATMPs have been included on the EUnetHTA Prioritization List, which indicated that these products are highly likely to be eligible for joint HTA assessment after the applications of market authorization are submitted to EMA (Table 5). Moreover, a major step toward joint HTA at the EU-wide level was seen in June 2021, with a political deal between the EU council and European Parliament being released (78). The new rules proposed that developers of health technology will only need to submit information, data and other evidence required for HTA once at the EU level. After enforcement, it is expected to facilitate access to innovative drugs for patients, reduce the administrative burdens especially for small companies, and inform better decision-making about price and reimbursement for national health authorities (78).

**Table 5:** ATMPs that are included on EUnetHTA prioritization list.

**INN**	**Product types**	**Structure**	**Manufacturers**	**Indication**	**Date of prioritization**
Remestemcel-L	Cell therapy	*Ex vivo* culture-expanded adult human mesenchymal stromal cells	Mesoblast	Crohn's disease	EPL 2.0 (July 2019)
Rexmyelocel T	Cell therapy	Autologous bone marrow-derived mononuclear cells	Rexgenero	Critical limb ischemia in patients with diabetes mellitus	EPL 1.0 (November 2018). Company has expressed willingness to participate in this Joint Assessment at the time of EMA submission
Rocapuldencel-T	Cell therapy	Autologous immunotherapy prepared from mature monocyte-derived dendritic cells	Argos	Metastatic renal cell cancer	EPL 2.0 (July 2019)
Valoctocogene roxaparvovec	Gene therapy	Adeno-associated viral vector (AVV)-5 gene therapies	Biomarin	Treatment of hemophilia A	EPL 2.0 (July 2019)
Elivaldogene autotemcel	Gene therapy	Autologous CD34+ hematopoietic stem cells (HSCs) transduced with lentiviral vector Lenti-D encoding the human ABCD1 cDNA	BlueBird Bio	Cerebral adrenoleukodystrophy	EPL 1.0 (November 2018). In dialogue with company concerning participation
ECCS-50	Cell therapy	Adipose-derived regenerative cells-based Habeo cell therapy	Cytori Therapeutics	Moderate to severe hand dysfunction due to scleroderma	EPL 1.0 (November 2018) Establishing contact with company
Lifileucel	Cell therapy	Autologous tumor infiltrating lymphocytes	Iovance	Malignant melanoma, advanced melanoma	EPL 2.0 (July 2019)

*EPL, EUnetHTA prioritization list*.

At the Member State level, one of the earliest examples of multicounty collaboration is the BeNeLuxA Initiative, now consisting of HTA bodies from Belgium, Netherlands, Luxembourg, Austria, and Ireland. Regarding the ATMP-related activities, BeNeLuxA has started the joint HTA for Zolgensma^®^ in May 2020 (79). The joint HTA report for Zolgensma^®^ was released in April 2021, which concluded that Zolgensma^®^ was not considered for reimbursement unless cost-effectiveness could be improved relative to existing therapies (80). After a joint price negotiation between Belgium, Ireland and the Netherlands that was initiated in July 2021, Zolgensma^®^ was reimbursed for two specific groups of children and will be available in all three countries this year (81). This is highlighted as the first time that these three countries have reached an agreement on the price of a drug. In Nordic countries, Finland, Norway, Sweden (FINOSE), established in 2018, is a collaborative initiative to make joint clinical and economic assessment between HTA bodies in Finland, Norway, and Sweden. Zynteglo^®^ is the third product (first ATMP) being evaluated by FINOSE, with an indicated ICER ranging from SEK 17,61,000 (1,75,300 Euros) to SEK 21,37,000 (212.730 Euros) (82). This result will be utilized for the following joint price negotiation between five Nordic countries including Denmark, Ireland and three FINOSE members, remarking the first drug to be jointly negotiated through Nordic collaboration (83). Another collaboration initiative engaging 11 European countries (including France as an observer) is a Valletta Declaration, which was formed in May 2017 and identified CAR-T as one of the priorities (84).

However, it should be recognized that, despite being ambitious, no product has been jointly assessed through a Valletta Declaration until now. Moreover, the absence of an official webpage of this group makes it difficult to track its progress (85). The contributing factors for stagnation could include language barriers, misaligned methodology for evidence assessment, diverse healthcare structures, and varying economic capability across countries (86). These challenges are also imperative in other cooperative organizations, such as EUnetHTA and BeNeLuxA. This is mirrored in the fact that the coordination process was proven to be complex and lengthy, and misalignments were indicated in the final price and reimbursement decisions made across involved member countries (87). Other unanswered questions include how to guarantee confidentiality, whether it will aggravate the existing inequity between wealthier countries (e.g., Nordic countries) and others, and how to harmonize the dispersed multi-nation activities with the centralized EU-wide activities (EUnetHTA) (85, 87). Kanavos et al. proposed that more clarifications on the HTA cooperation framework are needed, in terms of the definition of values, the quality of evidence appropriate for evaluation, the acceptance of real-world evidence, and the approaches to safeguard the consistency of relative evidence assessment across countries (77). Undoubtfully, to ensure that joint activities could really build synergies for streamlining the HTA process and accelerating patient access of ATMPs, further research is awaited to explore the appropriate governance approaches and clarify the working structure to address the aforementioned issues (86).

## Conclusion

**Figure 1:**
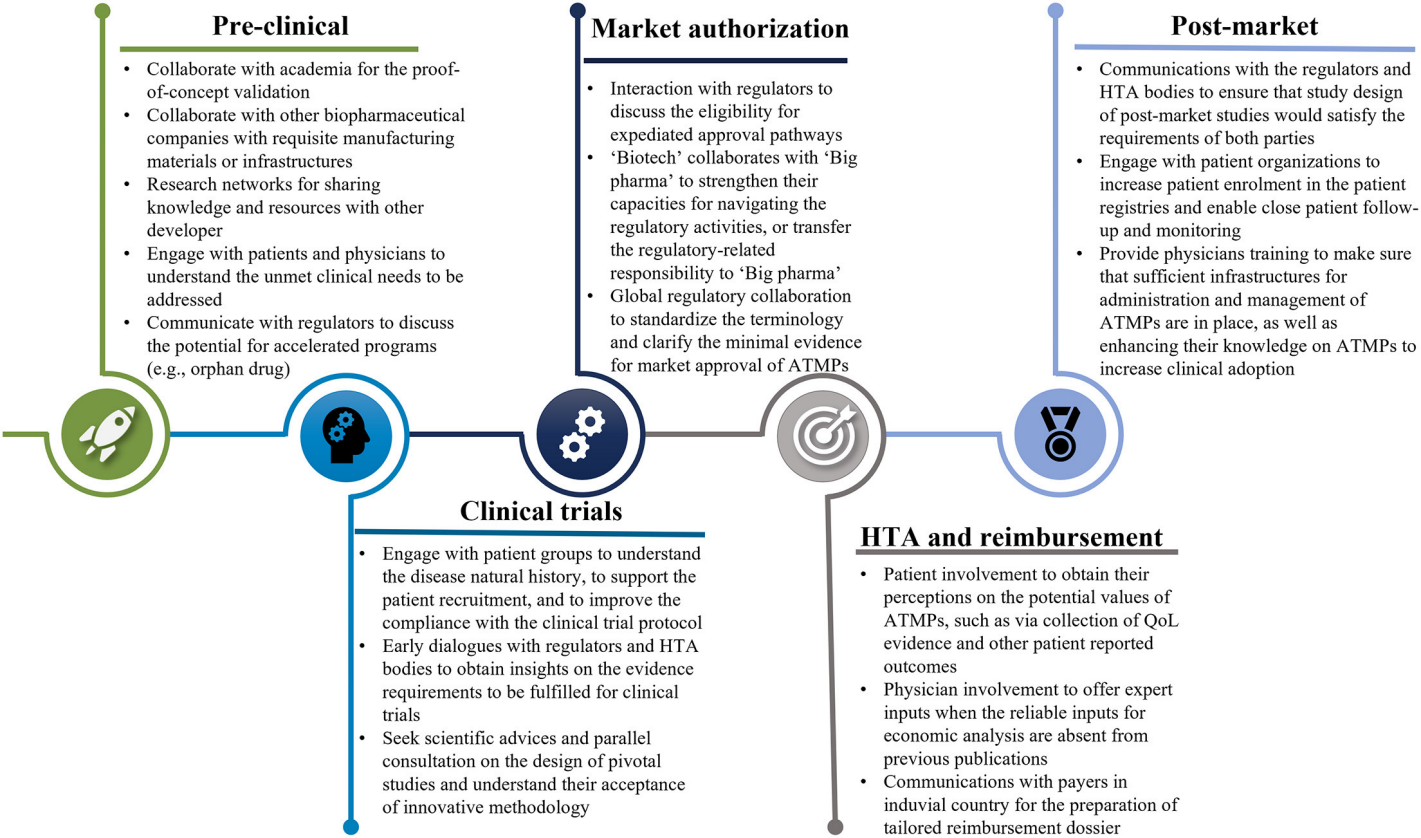
The collaborations with all stakeholders throughout the development of ATMPs. ATMPs, advanced therapy medicinal products.

**Figure 2:**
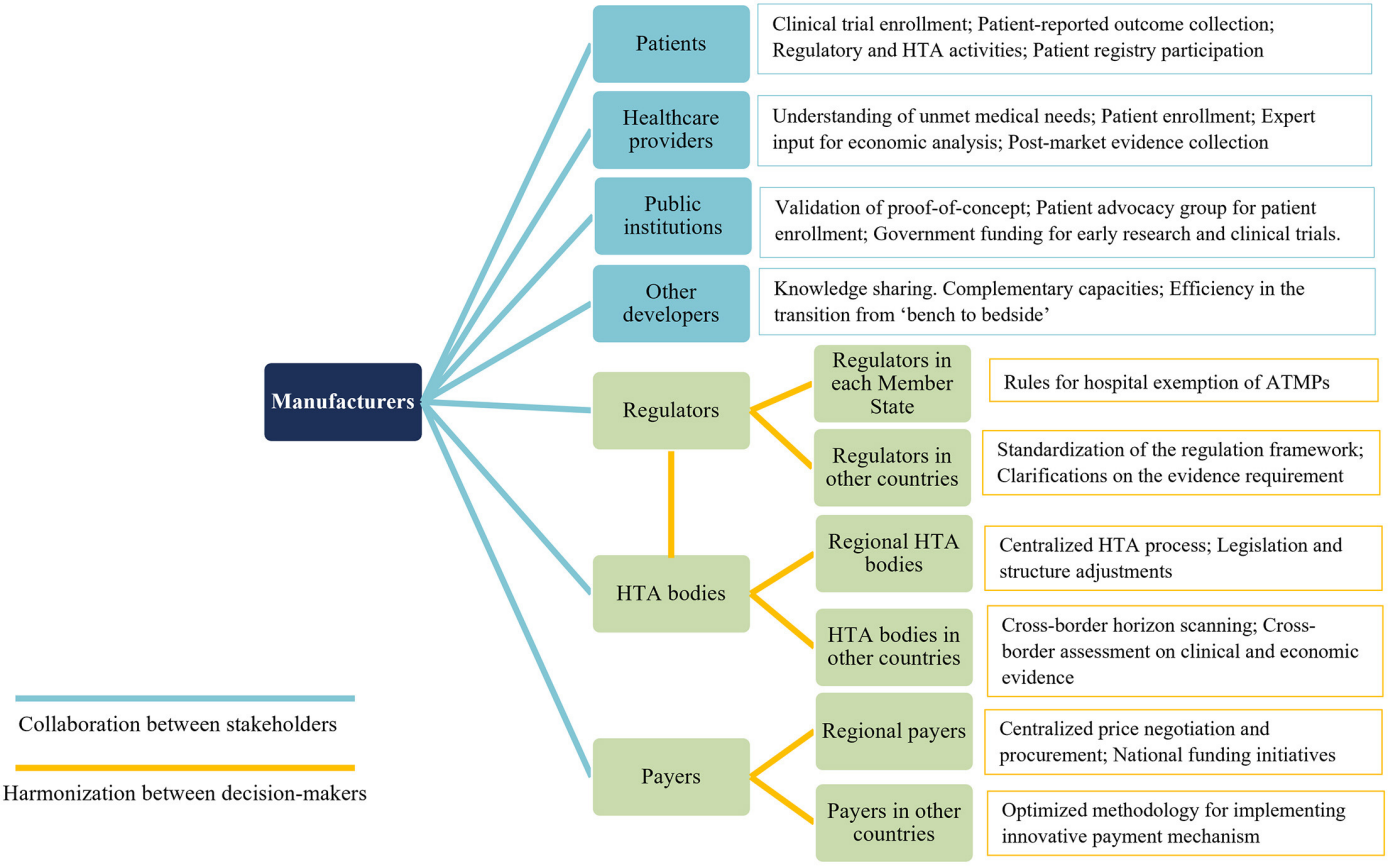
Key points for future collaboration and harmonization related to ATMPs. ATMPs, advanced therapy medicinal products.

Collaborative efforts from all relevant stakeholders will be paramount to overcome the substantial challenges existing in the research, manufacturing, clinical development, market authorization, HTA, pricing, reimbursement, and post-launch evidence collection of ATMPs (Figures 1, 2).

From the perspectives of manufacturers, collaboration could be strengthened in the aspects of (1) cooperation with other researchers or developers through partnership agreements or coordinated research network to obtain complementary capacity and promote knowledge sharing; (2) engagements with patients and healthcare providers to highlight their contributions throughout the whole development lifecycle, and (3) interactions with regulators and HTA bodies, preferably, through parallel consultation, to be better informed regarding the evidence requirements must be satisfied to secure positive recommendations.

From decision-markers' perspectives, international harmonization could be enhanced in the aspects of (1) alignments at the EU level on the requirements of ATMPs acceptable for HE; building patients registries interoperated in multiple countries; standardizing the regulation framework of ATMP; (2) joint HTA between national and regional level, as well as at multi-nation level, to improve efficiency, harness bargaining power, and narrow patient access gaps in different regions and countries.

To conclude, multi-stakeholder collaboration is paramount for manufacturers to strengthen the research and development capacity, and promote early patients access while ensuring that varying interests and expectations of all relevant stakeholders have been sufficiently balanced. Harmonization among decision-makers on the other hand, plays critical roles in reinforcing consistency and improving efficiencies of the regulatory and HTA process (Figure 1). Both elements need to be stressed to achieve timely patient access and to realize the potentials of promising ATMPs.

## Author Contributions

MT conceived the design of this review and drafted the table of contents. TQ wrote the entire manuscript. TQ, SL, and YW contributed to the literature searches and abstract drafting. CD and BB contributed to the section efforts from decision-makers to enhance international harmonization. All authors contributed to the article and approved the submitted version.

## Conflict of Interest

The authors declare that the research was conducted in the absence of any commercial or financial relationships that could be construed as a potential conflict of interest.

## Publisher's Note

All claims expressed in this article are solely those of the authors and do not necessarily represent those of their affiliated organizations, or those of the publisher, the editors and the reviewers. Any product that may be evaluated in this article, or claim that may be made by its manufacturer, is not guaranteed or endorsed by the publisher.
